# Validation of open-source deep learning segmentation tools for automated glioma volumetry: a narrative review of Dice scores, workflow efficiency, and clinical RANO 2.0 implementation

**DOI:** 10.3389/fneur.2026.1797487

**Published:** 2026-07-29

**Authors:** Marek Slachta, Matej Halaj, Klara Balazova, Ondrej Kalita, Lumir Hrabalek, Eva Cechakova, Pavla Kudlova

**Affiliations:** 1 Department of Neurosurgery, University Hospital Olomouc and Faculty of Medicine, Palacký University Olomouc, Olomouc, Czechia; 2 Department of Biomedical Engineering, University Hospital Olomouc, Olomouc, Czechia; 3 Department of Health Care Science, Faculty of Humanities, T. Bata University in Zlin, Zlin, Czechia; 4 Department of Radiology, University Hospital Olomouc and Faculty of Medicine, Palacký University Olomouc, Olomouc, Czechia

**Keywords:** brain tumor, deep learning, glioma segmentation, MRI, RANO 2.0, volumetry

## Abstract

**Background:**

Segmentation enables extraction of quantitative imaging features to enhance glioma diagnosis by volumetric measurements and treatment response assessment. This narrative review evaluates open-source software for glioma segmentation and alignment with Response Assessment in Neuro-Oncology (RANO 2.0) volumetric criteria.

**Approach:**

In this narrative review, we evaluated thirteen open-source tools selected for multimodal MRI sequence support (T1W, T1CE, T2W, FLAIR), performance on public datasets (BraTS Challenge), and applicability to RANO 2.0 volumetry. Assessment included Dice scores, workflow efficiency, advantages, limitations, and clinical translation potential.

**Results:**

Tools achieved Dice scores 0.73–0.92 for tumor subregions. Despite high analytical validation, clinical utility is limited: 85% of treatment response studies have bias risk in patient selection per QUADAS-2 appraisal. Critically, as of November 2024, no automated tools have been formally validated specifically against RANO 2.0 criteria, despite their 2023 emphasis on volumetric standardization.

**Conclusion:**

Open-source segmentation tools show promise for standardizing glioma volumetry with emerging tools (GlioMODA, AutoRANO) explicitly targeting RANO 2.0-compatible volumetric assessment. Hybrid approaches combining open-source innovation with commercial clinical integration could optimize clinical translation.

## Highlights

- Deep learning-based segmentation tools enable automated, reproducible glioma volumetry that improves upon manual and semi-automated methods, supporting objective treatment response assessment under RANO 2.0 criteria.
- Advanced AI-based segmentation tools such as nnU-Net achieve high accuracy and reproducibility in tumor delineation, making it possible to obtain the more objective volumetric measurements needed to apply the updated RANO 2.0 criteria.
- Open-source segmentation tools are innovative and accessible but face challenges including generalization across institutions, sensitivity to incomplete MRI protocols, and lack of prospective clinical validation; commercial solutions provide clinical integration and certification but are costly.
- Future developments will include federated learning approaches for multi-institutional collaboration, tools with explicit RANO 2.0 volumetric targeting (e.g., GlioMODA, AutoRANO), and integration of segmentation with clinical data management for comprehensive response assessment.

## 1 Introduction

Glial tumors are among the most common primary brain tumors in adults, with high rates of mortality and morbidity (1). Current treatment strategies involve maximally radical surgical resection, histopathological examination with molecular-genetic typing following the 2021 World Health Organization (WHO) classification of central nervous system tumors, and oncotherapy. Magnetic resonance (MRI) is pivotal both for diagnosis and for monitoring disease progression (2, 3). Automated analysis of MRI data makes it possible to obtain objective radiomics markers for assessing pre- and postoperative tumor volume, evaluating disease progression, and monitoring treatment efficacy (4–7). Software-based processing of imaging data is mathematically precise and enables targeted analyses that were previously impossible because they focus on changes that are often below the threshold of human detection.

A crucial step in volumetric analysis is segmentation, which identifies regions of interest (ROI) for extracting characteristic tissue features – notably, shape and texture - corresponding to individual components of tumor expansion.

The RANO 2.0 criteria were introduced to standardize response assessment for both high- and low-grade gliomas (8). Since their recent update, they treat volumetric measurements as being equivalent to conventional ones and also support further integration of non-invasive techniques, opening the door to greater use of radiomic methods (9, 10). A parallel comprehensive systematic review of automated longitudinal treatment response assessment by Shi et al., 2025 (11) evaluated 20 published studies using machine learning for brain tumor response classification. Notably, despite RANO 2.0 criteria being introduced in 2023 with explicit volumetric measurement emphasis, no studies had yet implemented automated assessment specifically validated against these updated criteria as of November 2024. This temporal gap highlights a critical opportunity: the thirteen open-source tools evaluated in the present review, particularly nnU-Net with their volumetric capabilities (Dice 0.89 and interclass correlation coefficient 0.92–0.99 respectively), position open-source software as candidates for RANO 2.0-compatible longitudinal assessment pipelines. Moreover, there are indications that with the latest classifications of glial tumors, appropriately trained deep learning tools could potentially support non-invasive molecular characterization, especially in high-risk patients (10, 12–14). However, prospective multicenter validation remains essential before clinical deployment.

Radiomics is being applied to gliomas in many ways. Segmentation models have been shown to enable more accurate differentiation of radiation necrosis from tumor recurrence, which is vital for subsequent clinical decision-making (15). Additionally, radiomics has been combined with radiogenomics to uncover the genomic basis of tumor heterogeneity, and radiomics models are being trained to predict tumor molecular markers such as the presence of isocitrate dehydrogenase mutations (14, 16). Recent studies have also emphasized the value of deep learning (DL) and ML software for analyzing gliomas after complex neurooncological treatment in order to evaluate tumor residue after surgery and potentially subsequent oncological treatment (4, 17, 18).

### 1.1 Segmentation of ROI

Before segmentation of the tumor and other ROI such as perifocal edema or non-contrast-enhancing tumor infiltrating components, the input data must be preprocessed. Most software tools have established preprocessing workflows that are already configured in their algorithms, enabling automated optimization of resolution and cropping of source data to the brain area only (so-called “skull-stripping”) before segmentation of ROI. Segmentation is a key step in volumetry that influences the quality of the extracted data and its subsequent processing (13, 14).

Segmentation was traditionally performed using manual or semi-automated methods, which are unfortunately very time-consuming and prone to significant inter-observer variability. However, the development of machine learning algorithms in recent years has enabled a shift toward fully automated, reproducible, and rapid segmentation. For example, in an earlier study conducted at our institution, semi-automatic segmentation of glioblastoma based on processed T1-weighted post-contrast sequences (i.e., T1CE) required around 45 min of work (19). Conversely, in our current projects that use more advanced software, workflow improvements have reduced the time needed for segmentation to around 3 min on average, and multiple MRI sequences can be processed in this time. While this improvement partly reflects greater skill on the part of the experts performing the segmentation, it should be noted that our processing times did not differ greatly from those reported by other researchers and can thus be considered to be reasonably representative (6, 20).

The Dice score, which is based on the agreement between an ML system and expert human evaluation, is frequently used as an objective measure of the reliability and performance of ML models in tasks such as image segmentation. It takes values between 0 and 1, with 0 indicating no overlap between the ML model’s output and the ground truth data (i.e., a human expert’s assessment) and 1 indicating perfect overlap. Open-source models for glioma segmentation such as nnU-Net, and 3D U-Net achieve Dice scores of 0.85–0.92 (13, 14).

Multimodal MRI sequences (T1W, T1CE, T2W, or FLAIR) are often used to identify individual parts of gliomas, including viable and necrotic tumor tissue, edema, the peritumoral infiltrating zone, and non-contrast-enhancing or contrast-enhancing areas of the tumor. Segmentation is based on “learned” data from datasets that may be public or held privately within individual research institutions. These datasets contain annotated regions based on expert consensus, and the software learns to recognize individual patterns in the associated MRI scans from their presentation. To date, 28 datasets from the period 2005–2025 have been released to the professional public, containing 62,019 MRI examinations from 5,515 patients (21). The most well-known of these datasets are those of the BraTS Challenge (Brain Tumor Segmentation Challenge), whose participants compete annually to segment sample data from around the world in order to refine their ML models and eliminate segmentation errors or ambiguities (22). The most recent BraTS Challenge in 2024 focused on post-treatment datasets and improving model generalization for real clinical scenarios (23).

### 1.2 Clinical questions driving volumetric assessment in glioma

Before evaluating specific software, it is worth restating why accurate volumetric segmentation matters in glioma management, since this directly determines which technical properties are clinically acceptable. The introduction of RANO 2.0 has formalized volumetric criteria as the primary response endpoint for both high- and low-grade gliomas (8). A ≥ 50% increase in contrast-enhancing tumor volume or a ≥ 40% increase in T2/FLAIR non-enhancing lesion volume now defines progressive disease, while a ≥ 50% decrease in enhancing volume defines partial response (8). Bidimensional measurements remain accepted but are increasingly considered insufficient for irregularly shaped or infiltrative lesions, and prospective trials of mutant IDH inhibitors have shown that volumetric endpoints detect treatment effects that bidimensional criteria miss (9).

From a clinical standpoint, not all segmentation errors are equivalent. Random errors that average out across serial scans degrade single-timepoint Dice scores but may have limited impact on longitudinal response classification. Systematic errors—for example, consistent over- or under-segmentation of the enhancing rim, or differential behavior in the presence of hemorrhage, post-surgical blood products, or steroid-induced enhancement—are far more problematic, because they bias volume trajectories in the same direction across timepoints and can push patients across the RANO 2.0 thresholds without any true biological change. Errors at the enhancing/non-enhancing interface are particularly clinically relevant, as this boundary defines both response classification and the target volume for radiotherapy planning.

The clinical relevance of each segmented compartment also differs by tumor type and disease phase. For high-grade contrast-enhancing tumors, the enhancing component is the primary target of response assessment, the resection cavity is critical for early post-operative evaluation (where residual enhancing tumor must be distinguished from blood products and reactive enhancement), and the FLAIR/T2 non-enhancing component informs assessment of treatment effect, pseudoprogression, and infiltrative extension. For low-grade and IDH-mutant gliomas, the FLAIR/T2 component is the dominant clinically actionable signal, and segmentation tools optimized for contrast-enhancing disease may underperform here. Peritumoral edema, while routinely segmented in BraTS-style pipelines, has limited independent clinical decision value in most response-assessment contexts.

**Figure 1:**
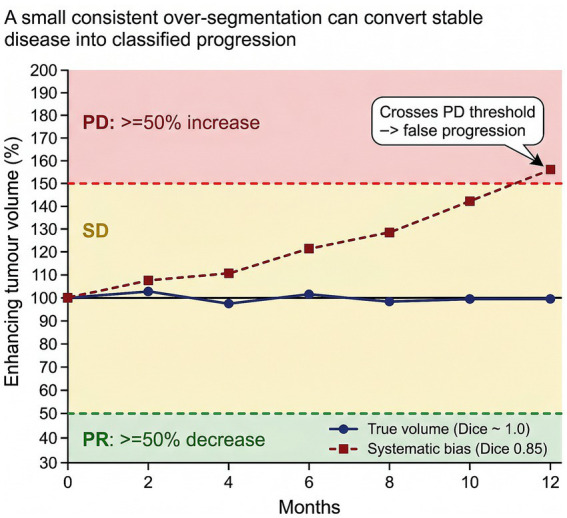
RANO 2.0 volumetric thresholds and impact of systematic segmentation bias.

These considerations have direct implications for how segmentation tools should be evaluated. A high single-timepoint Dice score is necessary but not sufficient: longitudinal repeatability (typically quantified by intraclass correlation coefficient on test–retest or double-baseline data), robustness across scanners and acquisition protocols, sensitivity for small residual lesions in the post-operative cavity, and behavior on the enhancing/non-enhancing interface are equally important. The framework *Metrics Reloaded* recently formalized this point for biomedical image analysis more broadly, recommending problem-specific metric selection rather than reliance on Dice alone (24). With this clinical framing in mind, the remainder of this review evaluates available tools against these criteria, drawing attention to which clinical questions each tool actually answers (Figure 1).

## 2 Methods

This narrative review evaluates thirteen open-source segmentation tools for glioma volumetry, selected based on criteria including open-source availability, relevance to brain tumor imaging (e.g., support for multimodal MRI sequences like T1W, T1CE, T2W, FLAIR), performance in public datasets (e.g., BraTS Challenge), and applicability to clinical scenarios such as RANO 2.0 volumetry. Tools were identified through literature searches (PubMed, GitHub repositories) and some of them were subjected to institutional testing at the University Hospital Olomouc.

We assessed datasets, Dice scores (from published validations), advantages, limitations, and implications for glioma management. The search covered publications from 2005 to January 2026, using search terms including “glioma segmentation,” “brain tumor radiomics,” “open source,” “deep learning segmentation,” and “RANO volumetry.” Inclusion criteria required tools to be (1) open source and freely available, (2) applicable to multimodal brain MRI data, (3) documented in peer-reviewed literature or established repositories, and (4) relevant to glioma segmentation or radiomic feature extraction. Tools were excluded if they were proprietary, lacked documentation for brain tumor applications, or had no published validation data. General-purpose frameworks such as MONAI and DeepMedic were included in revised version because, despite not being glioma-specific tools, they have been extensively validated on BraTS glioma datasets and represent important components of the current segmentation landscape. No new patient data were analyzed; all evaluations draw from cited sources and hands-on testing with anonymized institutional MRI scans (per protocols, no IRB approval required). It should be noted that the Dice scores reported for individual tools originate from different validation studies using heterogeneous datasets, lesion size distributions, and tumor region definitions, which limits direct cross-tool comparability. Readers should interpret these values as indicative of each tool’s performance within its specific validation context rather than as directly comparable benchmarks.

**Figure 2:**
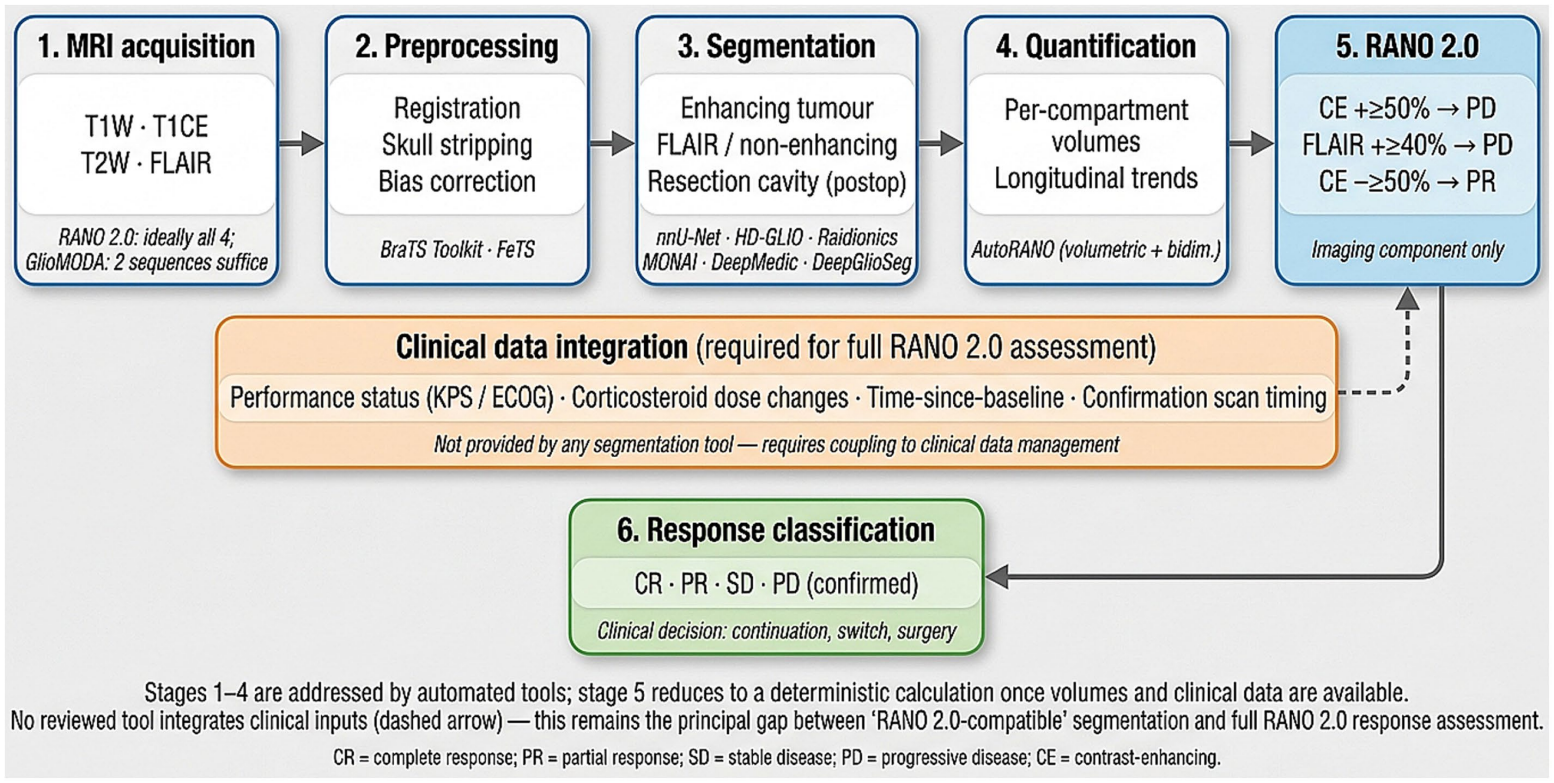
Automated glioma volumetry pipeline with reviewed tool positioning.

This review builds upon the framework established by a concurrent systematic review of ML-based automated longitudinal treatment response assessment which evaluated 20 studies using standardized quality assessment criteria (QUADAS-2) (11). While the Shi et al., 2025 (11) review focused on longitudinal response classification (determining progression, stable disease, partial response, or complete response), the present review emphasizes the foundational segmentation tools that enable such assessments. The Thirteen tools evaluated here represent the software infrastructure underlying the methodologies surveyed in the broader treatment response literature (Table 1, Figure 2).

**Table 1:** Comparative summary of reviewed open-source glioma segmentation tools.

Tool	Architecture/Approach	Segmentation target	Reported dice (validation context)	Training dataset	Postop capability	Incomplete MRI protocol	Longitudinal repeatability (ICC)	RANO functionality	GUI	Clinical maturity
ITK-SNAP	Semi-automatic (manual + region growing)	User-defined ROI	N/A (manual)	None	Yes (manual)	Yes	None	None	Yes	Research
3D Slicer	Modular platform; extension-based DL	User-defined ROI; GBM	0.80–0.84 (GBM comparative)	TCGA-GBM, BraTS (extensions)	Yes	Partial	None	None	Yes	Research
nnU-Net	Self-configuring U-Net	WT, TC, ET, cavity	0.77–0.89 (BraTS, postop multi-center)	BraTS	Yes (cavity Dice 0.81)	Limited	Not reported	None	No	Research; multi-center tested
Raidionics	nnU-Net-based	Pre/postop tumor, meningioma, mets	0.85 (preop); 0.41 (early postop)	Multi-institutional incl. BraTS	Yes (reduced perf.)	Limited	Not reported	None	Yes	Research
HD-GLIO	nnU-Net-based	Enhancing + FLAIR tumor	0.86 (CE >1 cm³); 0.40 (<1 cm³); 0.79 (FLAIR)	Multi-center (Heidelberg)	Yes	No (4 modalities)	Implicit (Kickingereder 2019)	Volumetric only	No	Research; multi-center validated
BraTS Toolkit	Ensemble fusion of BraTS-Challenge models	WT, TC, ET	Depends on fusion configuration	BraTS	No specific postop training	No (4 modalities)	Not reported	None	Partial (preproc only)	Research
TumorPrism3D	Semi-auto + AI-assisted; PyRadiomics integration	Pre/postop tumor	0.83–0.91 (pilot)	Not specified	Yes	Unclear	Not reported	None	Yes	Research; not publicly available at writing
DeepMedic	Multi-scale 3D CNN	WT, TC, ET	0.74–0.85 (BraTS + external, Pemberton 2023, 12 hospitals)	BraTS 2017/2021	Limited	No (4 modalities)	Not reported	None	No	Research; higher outlier rates than nnU-Net externally (8.75% vs 3.65% DSC; 36.21% vs 2.65% HD)
MONAI Auto3DSeg	Framework (SegResNet, Swin UNETR)	WT, TC, ET	~0.92/0.89/0.86 (BraTS 2021)	BraTS, multiple	Yes	Configurable	Not reported	None	No	Research framework; top-tier BraTS performance
FeTS	Federated ensemble (nnU-Net, DeepMedic, DeepSCAN)	WT, TC, ET	0.73/0.76/0.75 (FeTS 2024)	BraTS 2021 multi-site	Limited	No (4 modalities)	Not reported	None	Yes	Research; addresses multi-center validation barrier
GlioMODA	nnU-Net-based; protocol-robust	Enhancing + whole tumor	Not stat. diff. from manual (T1CE+FLAIR only)	BraTS 2021	Not externally validated	Yes (T1CE + FLAIR)	Not reported	Explicit volumetric targeting	No	Research; emerging RANO 2.0 focus
AutoRANO	DL pipeline + automated measurement	FLAIR + enhancing	High agreement vs. manual (ICC 0.915–0.965)	4-institution preop (n=843); single-center longitudinal (n=54 GBM)	Yes (single-center)	Limited	Yes (ICC 0.977–0.991)	Bidimensional + volumetric RANO	No	Research; most RANO-aligned of reviewed
DeepGlioSeg	CNN-Transformer hybrid (CTPC)	WT, TC, ET	Competitive on BraTS (dataset-dep.)	3 BraTS + 1 private	Not reported	No (4 modalities)	Not reported	None	No	Research

Reported Dice scores originate from heterogeneous validation studies (different datasets, tumor-region definitions, and lesion-size distributions) and are not directly comparable across tools; column entries should be read as descriptive of each tool's validation context. ICC, intraclass correlation coefficient; WT, whole tumor; TC, tumor core; ET, enhancing tumor; CE, contrast-enhancing.

## 3 Interactive platforms for clinical use

### 3.1 ITK-SNAP (Penn image computing and science laboratory (PICSL), University of Pennsylvania, USA)

ITK-SNAP is a software tool for semi-automatic segmentation of multimodal images. It was first described in NeuroImage (2006) and is not primarily trained (25). It is used for manual segmentation in studies analyzing BraTS data. Consequently, it has no Dice score.

*Open source*: https://www.itksnap.org/pmwiki/pmwiki.php.

*Implications*: This tool’s manual focus implies that it reduces subjectivity in small-scale studies but limits its scalability for RANO 2.0 volumetry in clinical routines, potentially increasing bias in infiltrative gliomas.

### 3.2 3D slicer (Surgical Planning Laboratory, Brigham and Women’s Hospital, Harvard Medical School, USA)

3D Slicer is a platform for visualization and analysis of medical images. It was initially reported in Magnetic Resonance Imaging (2012) and was first applied to gliomas in a study on GBM volumetry (2013) (26, 27). In most studies that use it, it is trained on the TCGA-GBM or BraTS datasets, although it has extensions that support manual/semi-automatic segmentation without specific training, and models can be trained on local data (28). In a comparative study, 3D Slicer achieved Dice scores of 0.80–0.84 for glioblastoma segmentation (29).

*Open source*: https://www.slicer.org.

*Implications*: Its 3D visualization supports clinical research by integrating with printing, implying better therapy planning under RANO 2.0 for volumetric tracking.

## 4 Fully automated deep learning platforms for clinical use

### 4.1 nnU-net (Division of Medical Image Computing, German Cancer Research Center (DKFZ), Germany)

nnU-Net is an interface for automatic segmentation based on the U-Net architecture, which adapts to the presented dataset. Although nnU-Net was used by its developers in segmentation challenges (including BraTS) and documented in accompanying conference proceedings prior to formal publication, the journal paper was published in Nature Methods (2020) and is typically trained on the BraTS dataset in studies that use it (30, 31). In its most recent evaluation (conducted in 2024), it achieved a Dice score of 0.81 for the contrast-enhancing tumor component, 0.77 for edema, and 0.81 for the surgical cavity in postoperative MRI (32). When applied to preoperative images, it achieves even higher Dice scores: 0.89 for whole tumors, 0.80 for the central necrotic part, and 0.82 for the contrast-enhancing tumor component (33). Recent validations (2025–2026) extend nnU-Net to post-treatment and pediatric gliomas, with Dice scores maintaining 0.77–0.89 across datasets, including ensembles for low-grade glioma response prediction (13, 34).

*Open source*: https://github.com/MIC-DKFZ/nnUNet.

*Implications*: The high Dice score of nnU-Net (0.89) implies reduced inter-observer variability, supporting the volumetric component of RANO 2.0 assessment and the potential to avoid surgical treatment of infiltrative gliomas by improving detection of recurrence.

### 4.2 Raidionics (SINTEF digital, Norwegian University of Science and Technology (NTNU), Norway)

Raidionics is open-source software for pre- and postoperative segmentation of brain tumors, including meningiomas and metastases. It was first presented in Scientific Reports (2023) and has already been trained on a multi-institutional dataset that is drawn from public sources such as BraTS and represents diverse tumor types, including gliomas (35). Its mean Dice score is 0.85 (35).

*Open source*: https://github.com/raidionics/Raidionics.

*Implications*: It was trained using multi-institutional data, suggesting that it will display good generalizability in real-world RANO 2.0 applications involving post-op residue evaluation.

### 4.3 HD-GLIO (Department of Radiology, Heidelberg University, University of Bonn, Germany)

HD-GLIO is a framework for pre- and postoperative glioma segmentation that is built on a deep neural architecture. Notably, HD-GLIO is built on the nnU-Net framework, having been developed using nnU-Net’s self-configuring pipeline with glioma-specific training data; consequently, direct performance comparisons between HD-GLIO and nnU-Net should be interpreted with caution, as the two share the same underlying architecture. It was first presented in Lancet Oncology (2019) (4). Its algorithm uses DL for automated tumor segmentation and its Dice score for well-defined contrast-enhancing lesions can be as high as 0.87 (36).

*Open source*: https://github.com/CCI-Bonn/HD-GLIO.

*Implications*: Its effectiveness for larger lesions suggests that it may be useful in initial RANO assessments, but it is likely to be less useful in follow-up as it may overlook small residues. It should be noted, however, that HD-GLIO’s automated segmentation outputs can be exported and loaded into interactive platforms such as ITK-SNAP or 3D Slicer for manual refinement, which partially mitigates limitations in standalone workflow integration.

### 4.4 BraTS Toolkit (Department of Informatics, Technical University of Munich, Germany)

The BraTS Toolkit is a modular open-source tool designed for automated brain tumor segmentation, enabling easy use of advanced algorithms (especially from the BraTS Challenge) in clinical practice and research without requiring deep technical knowledge. Its software was first presented in Frontiers in Neuroscience (2020) (37). Users can choose between command-line control or a simple GUI.

*Open source*: https://github.com/neuronflow/BraTS-Toolkit.

*Implications*: Fusion improves accuracy.

### 4.5 TumorPrism3D

TumorPrism3D is a semi-automatic research program with a robust GUI that is optimized for manual and AI-assisted pre- and postoperative glioma segmentation. It has built-in capabilities to compute basic statistics, and its output is compatible with PyRadiomics, enabling subsequent radiomic extraction. It was first reported in the Journal of Imaging Informatics in Medicine (2024) (29). Unfortunately, it was not publicly available at the time of writing, so we cannot list its advantages or disadvantages. In a pilot study, it achieved Dice scores of 0.83–0.91 (29).

### 4.6 DeepMedic (Biomedical Image Analysis Group, Imperial College London, UK)

DeepMedic is a multi-scale 3D convolutional neural network designed for brain lesion segmentation. It was first described by Kamnitsas et al. in Medical Image Analysis (2017) and won the BraTS 2017 Challenge as part of an ensemble of multiple models and architectures (EMMA), combining DeepMedic with FCN and U-Net (38, 39). On the BraTS 2017 validation set, the ensemble achieved Dice scores of 0.90 (whole tumor), 0.80 (tumor core), and 0.73 (enhancing tumor). In a subsequent multicenter evaluation by Pemberton et al. (2023) across 12 hospitals worldwide, DeepMedic — when retrained with sparsified training on combined BraTS 2021 and PICTURE clinical data — achieved median Dice scores within the human inter-rater agreement range (0.74–0.85) on both internal and external test sets. However, DeepMedic showed substantially higher outlier rates than nnU-Net (8.75% vs. 3.65% by Dice score, 36.21% vs. 2.65% by Hausdorff distance), highlighting greater susceptibility to performance degradation on real-world data.

*Open source*: https://github.com/deepmedic/deepmedic.

*Implications*: DeepMedic’s higher outlier rate on external clinical data (8.75% by Dice score, 36.21% by Hausdorff distance vs. nnU-Net’s 3.65 and 2.65%) serves as a cautionary example for RANO 2.0-compatible deployment: tools may achieve clinically acceptable median performance yet produce unreliable individual segmentations in routine clinical settings, where outlier behavior matters for trustworthy volumetric response assessment. Its outputs can, however, be loaded into interactive platforms such as ITK-SNAP or 3D Slicer for manual refinement.

### 4.7 MONAI Auto3DSeg (NVIDIA and Open-Source Community)

MONAI (Medical Open Network for Artificial Intelligence) is an open source, PyTorch-based framework for deep learning in healthcare imaging, first described by Cardoso et al. (2022) (40). While MONAI is a general-purpose framework rather than a glioma-specific tool, its Auto3DSeg pipeline and pre-trained models (including SegResNet and Swin UNETR architectures) have achieved top-tier results on BraTS benchmarks. The NVAUTO team using MONAI Auto3DSeg achieved 1st place in three BraTS 2023 challenges (Brain Metastasis, Meningioma, BraTS-Africa) and 2nd place in Adult and Pediatric Glioma (41). On the BraTS 2021 validation set, MONAI-based Swin UNETR models achieved Dice scores of approximately 0.92 (whole tumor), 0.89 (tumor core), and 0.86 (enhancing tumor). MONAI also provides pre-trained brain tumor segmentation models available through NVIDIA NGC.

*Open source*: https://github.com/Project-MONAI/MONAI.

*Implications*: MONAI represents the current state-of-the-art framework approach to medical image segmentation. Its Auto3DSeg pipeline and challenge-winning performance position it as a strong candidate for RANO 2.0-compliant volumetric pipelines, particularly when combined with nnU-Net in ensemble configurations.

### 4.8 FeTS (federated tumor segmentation) (Center for Biomedical Image Computing and Analytics, University of Pennsylvania, USA)

FeTS is an open-source ecosystem for privacy-preserving brain tumor segmentation using federated learning. It was first described by Pati et al. in Physics in Medicine and Biology (2022) (42). The FeTS tool integrates three pre-trained BraTS challenge models (DeepMedic, DeepSCAN, and nnU-Net) and provides label fusion strategies to generate consensus segmentations. Built on the BraTS 2021 dataset (1,251 training cases, 219 validations, 570 test cases), the FeTS Challenge 2024 evaluated six teams, with the top-performing method achieving mean Dice score values of 0.73 (enhancing tumor), 0.76 (tumor core), and 0.75 (whole tumor). The FeTS tool includes a user-friendly GUI, preprocessing pipelines, and supports both inference from pre-trained models and collaborative federated training across institutions.

*Open source*: https://github.com/FETS-AI/Front-End.

*Implications*: FeTS addresses a critical barrier to RANO 2.0-compatible multi-institutional deployment: the need for models validated across diverse institutional data while maintaining patient privacy. Its federated approach may become essential for multicenter clinical trials requiring standardized volumetric assessment.

### 4.9 GlioMODA (Division of Medical Image Computing, DKFZ, Germany)

GlioMODA is a deep learning framework specifically designed for robust automated glioma segmentation across varied and incomplete MRI protocols. It was presented on Neuro-Oncology Advances (2026) and trained on the BraTS 2021 dataset (1,251 training, 219 testing cases), systematically evaluating performance across eleven clinically relevant MRI protocol combinations (43). Critically, GlioMODA demonstrated that a streamlined two-sequence protocol (T1-CE + T2-FLAIR) yielded volumetric differences versus manual ground truth that were not statistically significant for enhancing tumor (median difference 55 mm^3^, *p* = 0.157) and whole tumor (median difference −7 mm^3^, *p* = 1.0). Both trained models and source code are released as open source.

*Open source*: https://github.com/BrainLesion/GlioMODA/.

*Implications*: GlioMODA is one of the first tools explicitly aligned with RANO 2.0 volumetric requirements, demonstrating that clinically reliable glioma volumetry is achievable with a minimal two-sequence MRI protocol. This directly addresses the practical barrier of incomplete clinical MRI data.

### 4.10 AutoRANO (Martinos Center for Biomedical Imaging, Massachusetts General Hospital/Harvard Medical School, USA)

AutoRANO is a fully automated, open-source deep learning pipeline for brain extraction, glioma segmentation (FLAIR hyperintensity and contrast-enhancing tumor), and automated RANO measurements including both bidimensional diameters and volumetric quantification. It was first reported by Chang et al. in Neuro-Oncology (2019) (17). The pipeline was validated on 843 preoperative MRIs from four institutions and 713 longitudinal postoperative MRI visits from 54 glioblastoma patients. Automatically generated volumes demonstrated high repeatability (ICC 0.986 for FLAIR, 0.991 for CE tumor, 0.977 for AutoRANO) and high agreement with manual expert measurements (ICC 0.915–0.965 for volumes). Longitudinal change measurements showed ICC of 0.917–0.966 for volume changes and 0.850 for RANO measurement changes.

*Open source*: https://github.com/QTIM-Lab/AutoRANO.

*Implications*: AutoRANO is uniquely positioned for the imaging-derived components of RANO 2.0 as the only reviewed tool that automates both volumetric and bidimensional measurements; full RANO 2.0 response assessment additionally requires integration of clinical performance status and corticosteroid data. Its longitudinal validation and high ICC values suggest readiness for integration into clinical trial response assessment workflows, potentially reducing interobserver variability and adjudication rates.

### 4.11 DeepGlioSeg

DeepGlioSeg is a U-shaped CNN-Transformer hybrid architecture for multimodal glioma MRI segmentation. It was first reported in Frontiers in Oncology (2025) (44). The core innovation is the CTPC (CNN-Transformer Parallel Combination) module, which fuses local features from CNN branches with global dependencies from Transformer branches. The model employs a weighted loss approach to address tumor region imbalance. DeepGlioSeg was evaluated on three public BraTS datasets and one private clinical dataset, demonstrating competitive Dice scores with post-processing refinements (test-time augmentation and volume-constrained filtering). Source code is publicly available on GitHub.

*Open source*: https://github.com/smallboy-code/Brain-tumor-segmentation.

*Implications*: DeepGlioSeg represents the emerging trend of hybrid CNN-Transformer architectures in glioma segmentation. While not yet clinically translated, its multi-dataset validation and open-source availability make it a candidate for future RANO 2.0 volumetric pipelines, particularly for handling heterogeneous tumor morphologies.

## 5 Discussion

Throughout this review, ‘RANO 2.0-compatible’ or ‘RANO 2.0 volumetric requirements’ refer to tools that support the imaging-derived volumetric component of the RANO 2.0 criteria. Full RANO 2.0 response assessment additionally integrates clinical performance status, corticosteroid dose changes, and temporal criteria for confirmation of progression or response (8). No segmentation tool, however accurate, can be considered ‘RANO 2.0 compliant’ in isolation: a complete RANO 2.0 pipeline requires coupling of imaging segmentation to clinical data management. We use the ‘compatible’ terminology to make this distinction explicit.

Automatic glioma segmentation is a rapidly evolving field with significant potential to transform the management of glial brain tumors through quantitative analysis of medical imaging data. The open-source software tools reviewed here are flexible and freely accessible. Moreover, they generally lack formal clinical validation and regulatory approval, which complicates their integration into routine clinical workflows. While segmentation tools achieve high Dice coefficients (0.77–0.92), these values originate from different validation studies with heterogeneous test datasets, lesion size distributions, and evaluated tumor regions, limiting direct cross-tool comparisons. Furthermore, comprehensive assessment of their clinical utility is limited. A systematic review of 20 automated treatment response studies (11) determined that only 15% of studies met low-risk criteria for patient selection and index test design per QUADAS-2 (Quality Assessment of Diagnostic Accuracy Studies 2) appraisal. Specifically, 85% of studies had unclear or high risk of bias in patient selection, and 80% had similar concerns regarding reference standard definition. These findings underscore that high analytical validation (Dice scores) does not guarantee clinical validation or readiness for deployment. Most critically, as of November 2024, no ML-based tools had been formally validated against the updated RANO 2.0 criteria, despite their emphasis on volumetric standardization. However, 2025–2026 advancements, including AI-RAPNO recommendations for pediatric translation and AutoRANO-like tools for volumetric metrics, suggest maturing compliance (45, 46).

It is important to note that RANO 2.0 defines specific volumetric thresholds for disease progression states: a ≥ 50% increase in the volume of contrast-enhancing disease or a ≥ 40% increase in T2/FLAIR non-enhancing lesion volume constitutes progressive disease, while a ≥ 50% decrease in enhancing volume represents partial response. These thresholds require not only accurate single-timepoint segmentation but, more importantly, longitudinal consistency of measurements. A segmentation tool with a moderate Dice score that consistently tracks lesion volume changes over time may be more clinically useful for RANO 2.0-compatible volumetric assessment than a tool with a higher Dice score but poor longitudinal reproducibility. The Dice coefficient, as evaluated in this review, captures agreement at a single timepoint but does not assess whether segmentation errors are systematic or random across serial scans, which is the key determinant of reliable volumetric trend monitoring. Furthermore, small lesions with segmentation errors will yield disproportionately low Dice scores compared to large lesions with similar absolute inaccuracies, an important consideration when evaluating tool performance across heterogeneous tumor sizes encountered in clinical practice. Additionally, RANO 2.0 incorporates clinical factors such as corticosteroid use and clinical status into response assessment, which are not captured by imaging-based segmentation tools alone. A comprehensive RANO 2.0-compliant automated pipeline would therefore need to integrate imaging segmentation with clinical data management, including documentation of steroid dosage changes, to provide a complete response assessment.

Automatic segmentation frameworks such as nnU-Net achieve high Dice similarity coefficients (0.81 to 0.86) for glioma segmentation, surpassing many traditional methods (32, 33). However, despite their promise, open-source solutions have certain limitations. Their installation and optimization require technical expertise, they are sensitive to variability in imaging data (e.g., heterogeneity or missing MRI modalities), and they can produce segmentation errors in postoperative imaging scenarios. Finally, some open-source tools may exhibit limited generalizability and variable performance when applied to multicenter data, creating a risk of bias and problems like image quality issues if used in non-validated settings. Generalization across institutions and datasets represents a critical limitation. Shi et al., 2025 found that 60% trained and tested exclusively on post-operative data from single centers, while 40% used pre-operative BraTS datasets for training and post-operative clinical data for testing-creating a ‘domain gap’ that potentially inflates apparent performance. Only 15% of reviewed studies employed prospective data collection, and only 55% incorporated multi-institutional datasets (11). The open-source tools reviewed here similarly show varying performance on external datasets (e.g., nnU-Net Dice ranging from 0.77–0.89 depending on dataset and sequences), suggesting that institutional implementation requires site-specific validation rather than direct adoption of published metrics. To address these gaps, future studies should prioritize prospective multicenter designs with external validation datasets and explicit validation against RANO 2.0 criteria. Integrating DTI-based radiomics for infiltration mapping could further enhance recurrence prediction (47).

The set of tools reviewed here reveals an important trend toward RANO 2.0-specific functionality. GlioMODA demonstrates that reliable glioma volumetry is achievable with only two MRI sequences (T1CE and T2-FLAIR), directly addressing the barrier of incomplete clinical protocols. AutoRANO uniquely provides both volumetric and bidimensional RANO measurements with high longitudinal reproducibility (ICC 0.977–0.991), making it the most directly RANO 2.0-aligned tool reviewed. The FeTS framework addresses the complementary challenge of multi-institutional validation through federated learning, enabling privacy-preserving model training across sites. Meanwhile, hybrid CNN-Transformer architectures such as DeepGlioSeg and MONAI’s Swin UNETR represent the evolving state-of-the-art, with BraTS 2023 ensembles achieving Dice scores of 0.90 (whole tumor), 0.87 (tumor core), and 0.85 (enhancing tumor) (41). The multicenter comparison by Pemberton et al. (2023) remains the most rigorous cross-tool evaluation, confirming nnU-Net’s superior generalization (3.65% outlier rate vs. 8.75% for DeepMedic) (6). Collectively, these findings suggest that while no single tool currently satisfies all RANO 2.0 requirements, a combination of AutoRANO for measurement automation, GlioMODA or nnU-Net for robust segmentation, and FeTS for federated multi-institutional deployment could constitute a comprehensive RANO 2.0-compatible pipeline.

### 5.1 From segmentation to biology: error propagation in radiomic and radiogenomic pipelines

Segmentation is the first step of a multi-stage analytical pipeline that ultimately attempts to extract biological and prognostic information from imaging. Errors at the segmentation stage do not remain confined there: they propagate into downstream radiomic feature extraction and, by extension, into molecular prediction and radiogenomic models. A systematic review of 41 studies found that radiomic features are sensitive—to varying degrees—to image acquisition settings, reconstruction algorithm, preprocessing, and segmentation, with textural and shape features generally less reproducible than first-order statistics (48). The Image Biomarker Standardization Initiative (IBSI) has formalized feature definitions and provided reference values to mitigate inter-software variability, but adoption remains inconsistent across studies (49).

For glioma specifically, two consequences are clinically relevant. First, edge voxels at the tumor/edema or enhancing/non-enhancing interface carry disproportionate information density for infiltrative pattern characterization, meaning that small segmentation differences at these boundaries can substantially alter texture and shape features even when whole-tumor Dice is high. Surface Dice and Hausdorff distance capture boundary fidelity better than volumetric Dice and are preferable when downstream radiomic analysis is intended yet are reported for only a minority of the tools reviewed here. Second, radiomic models trained for molecular prediction—most prominently IDH mutation status and MGMT methylation—may show drift in sensitivity and specificity when the underlying segmentation pipeline changes between institutions. This implies that sharing trained radiogenomic models without sharing (or at least standardizing) the segmentation pipeline is unlikely to yield reproducible performance, and that multi-institutional radiogenomic validation should report not only feature extraction parameters but also the specific segmentation tool and version used.

These observations qualify the optimistic framing of automated segmentation as an enabler of precision oncology: such enablement requires the entire pipeline—not just segmentation—to be standardized and validated end-to-end. From the perspective of clinical translation, this argues for prospective validation studies that report segmentation, feature extraction, and molecular prediction performance jointly, rather than evaluating segmentation as an isolated technical task.

In contrast, commercial software platforms like Quibim Precision (Quibim, Spain) and NeuroQuant (Cortechs.ai, California, USA), offer integrated, user-friendly segmentation solutions with clinical certification by authorities such as the FDA as well as CE marking, workflow automation, and PACS system integration (50, 51). This facilitates their use in clinical settings, especially for the precise volumetric tumor assessments and recurrence monitoring called for by RANO 2.0. However, commercial platforms are often costly, proprietary, and less customizable, limiting accessibility for smaller research groups and institutions.

An important consideration that tempers the clinical applicability of radiomics is the limited robustness of radiomic features. A growing amount of literature has demonstrated that radiomic features are sensitive to variations in MRI acquisition parameters (e.g., field strength, sequence parameters, slice thickness), scanner manufacturers, reconstruction algorithms, and image preprocessing steps. These sources of variability can introduce significant feature instability, potentially undermining the reproducibility of radiomics-based models across institutions and longitudinal assessments. While standardization initiatives such as the Image Biomarker Standardization Initiative (IBSI) and harmonization techniques (e.g., ComBat) have been proposed, their adoption remains inconsistent. Consequently, the promise of clinical applicability of radiomics should be considered in light of these robustness challenges, and multicenter validation with harmonized acquisition protocols remains essential before routine clinical deployment.

### 5.2 Practitioners’ notes from single-center pilot testing

The following observations derive from informal hands-on testing of a subset of the reviewed tools at our institution. They were not obtained through a standardized, blinded, or quantitatively scored comparison, and they should be read as practical implementation notes rather than comparative evaluation. With that caveat, several patterns recurred across our pilots and may be useful for groups planning similar deployments. Tools with mature graphical user interfaces (ITK-SNAP, 3D Slicer, Raidionics) substantially lowered the entry barrier for clinical collaborators, but manual or semi-automatic workflows in ITK-SNAP and 3D Slicer remained too time-intensive for cohort-scale analyses, with manual T1CE-based glioblastoma segmentation requiring approximately 45 min per case in our earlier institutional work (19) versus approximately 3 min per case for current automated workflows—a difference broadly consistent with published reports (6, 20). nnU-Net produced the most accurate segmentations in our hands but lacked an integrated GUI for downstream visual verification and was constrained by the absence of GPU acceleration on ARM-based macOS workstations, which materially affected training (though not inference) feasibility. Raidionics provided a cohesive end-to-end interface and reliable preoperative segmentation across high- and low-grade gliomas, but occasionally segmented regions outside the intended ROI and did not always automatically identify T1W versus T1CE sequences, requiring manual sequence assignment that extended processing time. We emphasize again that these are single-center, non-quantitative observations and should not be over-interpreted; they are reported only to flag practical considerations that may not be apparent from published validation metrics alone.

**Figure 3:**
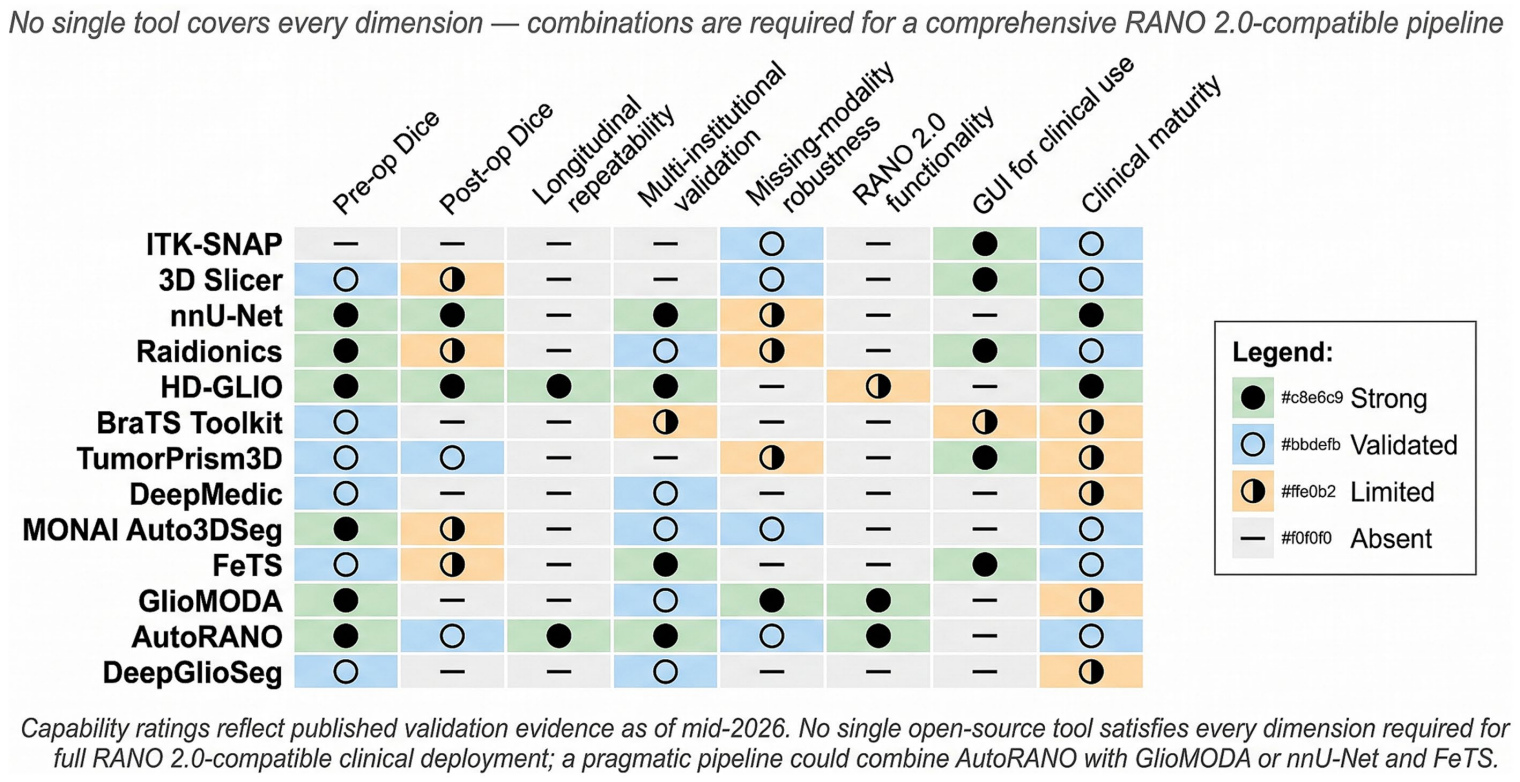
Capability heatmap across reviewed tools and clinical readiness dimensions.

Looking forward, automated glioma segmentation and volumetry show great potential for improving glioma diagnosis, treatment monitoring, and prognostication by combining advanced machine learning methods with multimodal imaging, clinical data, and integration with other -omics methods (e.g., genomics for IDH mutation prediction). To realize its full potential, future work should focus on rigorous clinical validation, standardization of methodologies, and integration with molecular and genetic tumor profiling. Such multidisciplinary development could enable personalized medicine approaches that will greatly improve patient outcomes (Figure 3).

## 6 Conclusion

In conclusion, while open source radiomics software fosters innovation and is broadly accessible to researchers, commercial platforms provide clinical readiness and regulatory compliance. They thus have complementary roles in advancing the field. Notably, tools such as GlioMODA and AutoRANO represent a new generation of open source solutions explicitly designed for RANO 2.0-compatible volumetry, while federated approaches like FeTS address the critical need for multi-institutional validation without compromising patient privacy. Future research should aim to develop hybrid solutions that combine the strengths of these tools to accelerate their translation from research to clinical practice. Given that no automated tools have yet been formally validated against RANO 2.0 criteria, this represents a critical near-term research priority. Prospective multicenter studies incorporating external validation datasets and explicit validation against the volumetric criteria of RANO 2.0 are now essential to move open source radiomics tools from research environments into clinical practice.
